# Preclinical Evidence of Nanomedicine Formulation to Target *Mycobacterium tuberculosis* at Its Bone Marrow Niche

**DOI:** 10.3390/pathogens9050372

**Published:** 2020-05-13

**Authors:** Jaishree Garhyan, Surender Mohan, Vinoth Rajendran, Rakesh Bhatnagar

**Affiliations:** 1 Laboratory of Molecular Biology and Genetic Engineering, School of Biotechnology, Jawaharlal Nehru University, New Delhi 110067, India; Jgarhyan@gmail.com (J.G.); mohan.surender@gmail.com (S.M.); 2 Department of Biochemistry, University of Delhi South Campus, Benito Juarez Road, New Delhi 110021, India; vinoth.avj@gmail.com; 3 Department of Microbiology, School of Life Sciences, Pondicherry University, Puducherry 605014, India; 4 Banaras Hindu University, Varanasi, Uttar Pradesh 221005, India

**Keywords:** BM-MSCs, Mtb, bone-homing, stem cell niche, latent tuberculosis, relapse, liposomes

## Abstract

One-third of the world’s population is estimated to be latently infected with *Mycobacterium tuberculosis* (Mtb). Recently, we found that dormant Mtb hides in bone marrow mesenchymal stem cells (BM-MSCs) post-chemotherapy in mice model and in clinical subjects. It is known that residual Mtb post-chemotherapy may be responsible for increased relapse rates. However, strategies for Mtb clearance post-chemotherapy are lacking. In this study, we engineered and formulated novel bone-homing PEGylated liposome nanoparticles (BTL-NPs) which actively targeted the bone microenvironment leading to Mtb clearance. Targeting of BM-resident Mtb was carried out through bone-homing liposomes tagged with alendronate (Ald). BTL characterization using TEM and DLS showed that the size of bone-homing isoniazid (INH) and rifampicin (RIF) BTLs were 100 ± 16.3 nm and 84 ± 18.4 nm, respectively, with the encapsulation efficiency of 69.5% ± 4.2% and 70.6% ± 4.7%. Further characterization of BTLs, displayed by sustained in vitro release patterns, increased in vivo tissue uptake and enhanced internalization of BTLs in RAW cells and CD271+BM-MSCs. The efficacy of isoniazid (INH)- and rifampicin (RIF)-loaded BTLs were shown using a mice model where the relapse rate of the tuberculosis was decreased significantly in targeted versus non-targeted groups. Our findings suggest that BTLs may play an important role in developing a clinical strategy for the clearance of dormant Mtb post-chemotherapy in BM cells.

## 1. Introduction

Tuberculosis (TB) remains one of the most common human diseases today, causing nearly two million deaths per year [1]. One-third of the global population is estimated to be latently infected with *Mycobacterium tuberculosis*, which is attributed to the ability of the tubercle bacillus to remain dormant in its protective niche unrecognized by the host immune system [1,2,3,4,5,6]. In post-chemotherapy patients, the recurrence of TB is very common due to endogenous reactivation leading to death [5,7,8,9,10]. It is imperative to find strategies that could effectively target and facilitate dormant *Mycobacterium tuberculosis* (Mtb) clearance to avert disease reactivation and associated mortality. 

CD271+bone marrow mesenchymal stem cells (BM-MSCs) serve as a novel host and a protective niche for non-replicating dormant *Mycobacterium tuberculosis* [11,12,13,14]. Mycobacterial bacilli remains intracellularly protected by standard anti-Tb drug treatment in mesenchymal stem cell populations, as shown in murine models and post-chemotherapy TB patients [11,13,14,15]. Das et al. demonstrated that in post-DOT (direct observed treatment) patients, a specific population of MSCs with CD271+ surface marker, harbor live nonreplicating Mtb bacilli [12]. The residual dormant Mtb population post-drug treatment may lead to endogenous reactivation and develop resistant forms [5,6,10,16,17,18]. We and others have shown that in murine models, prolonged anti-Tb drugs at standard dosages fail to clear the Mtb population inside CD271+BM-MSCs [13,14,19]. More importantly, despite 90 days of anti-Tb drug treatment in mice, CD271+BM-MSC-resident Mtb reactivates and leads to relapse [14]. We hypothesized that a BM-directed approach where anti-Tb drugs are delivered specifically to the bone microenvironment, can target Mtb inside BM-MSCs and reduce the relapse rate. 

We and others have shown that generic nanoparticle formulations impart protection and provide countermeasures against various infections [20,21,22,23,24,25,26]. Currently, to reduce off-target toxicity and enhance effective tissue treatment, organ-homing nanoparticles (NPs) are being used as drug-delivery vehicles [27,28,29,30,31]. Among other NPs, liposomes are the most versatile and are widely used in clinical settings because of their biocompatibility and controlled release profile [21,27,30,32]. Recent studies show that the bone microenvironment could specifically uptake surface-modified liposomes and PLGA NPs (poly (lactic-co-glycolic acid)) compared to other organs [33,34,35]. Although for TB infections, tissue-specific liposomes have been reported previously [22,36,37,38,39,40,41], a novel approach of bone homing liposomes has not been addressed yet. In this study, we employed a biphosphate molecule, alendronate (Ald) as target moiety and PEG to formulated bone-homing liposomes in order to deliver the standard anti-Tb drugs rifampicin (RIF) and isoniazid (INH) right to the bone microenvironment. We propose that drug encapsulated bone-homing PEGylated liposome (BTL) nanoparticles can clear the Mtb bacilli residing in CD271+BM-MSCs and reduce the relapse rate.

## 2. Results

### 2.1. Designing of Alendronate BTL-NPs and Their Characterization 

**Figure 1:**
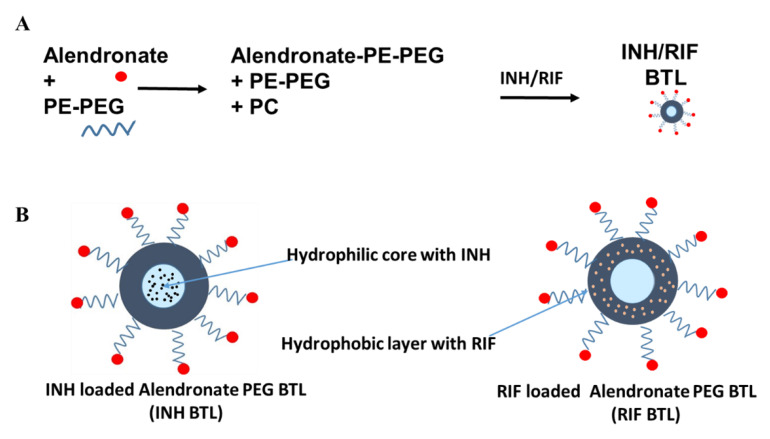
Designing of alendronate bone-homing PEGylated liposome (BTL) nanoparticles. (**A**) Alendronate and PE-PEG undergo chemical reactions for the binding of alendronate to form alendronate PE-PEG. Alendronate-PE-PEG is attached to the surface of liposomes. (**B**) Schematic of isoniazid (INH) and rifampicin (RIF) encapsulated alendronate PEG BTL-NPs. PC = Phosphatidyl-choline, CH_2_CH_2_N^+^(CH_3_)_3_; PE = Phosphatidylethanolamine, CH_2_CH_2_NH_3_^+^.

**Figure 2:**
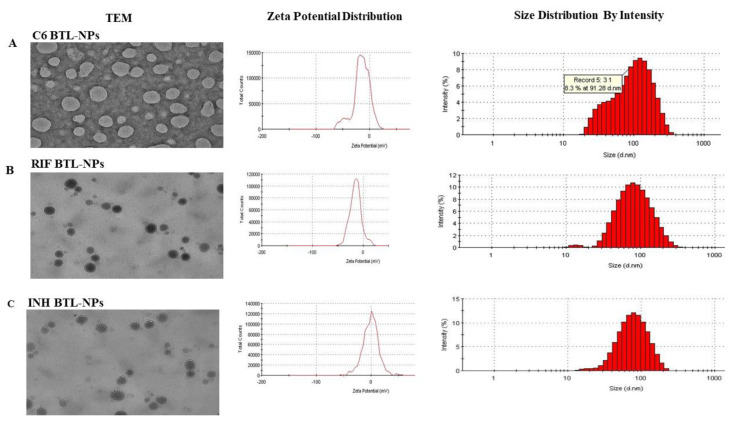
Characterization of liposomal formulations (TEM and size distribution of BTLs). Transmission electron microscopy (JEOL 2100F) images of all the three BTLs encapsulating C6, INH and RIF were performed. Dynamic light scattering and zeta potential were measured using Malvern Zetasizer. The above figure is a representative image for each liposomal formulation.

**Figure 3:**
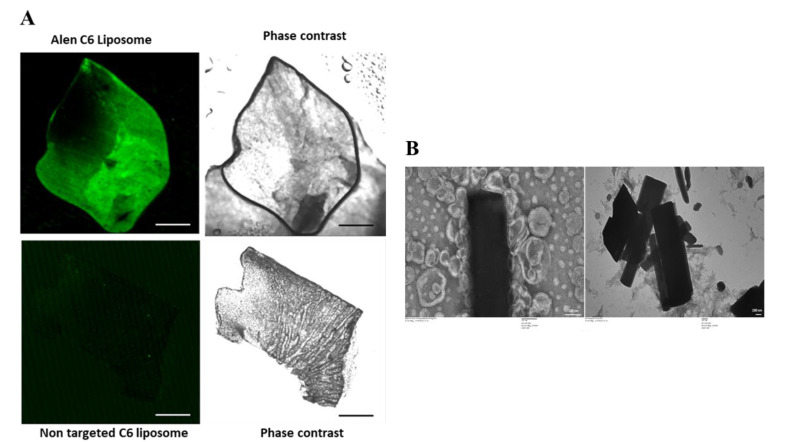
(**A**) Bone-chip binding assay of BTL-NPs. Bone fragments from femur were washed and incubated with alendronate BTLs encapsulating Coumarin6 (alen BTL C6) and non-targeted liposomes C6 (NT liposomes C6) for 30 min followed by washing and visualization through a confocal microscope (scale bar = 0.1 mm). (**B**) BTL’s affinity to Hydroxyapatite (HA)**.** For HA binding assay of BTL-NPs, HA microparticles were incubated with BTLs or NT liposomes. A 200 µL volume of 0.05 mg/mL HA was incubated with 40 µL of BTL or NT followed by rotation for 30 min at room temperature. The samples were washed 3 times gently and loaded on a TEM grid followed by staining and visualization with TEM (JEOL 2100F). Black rods are cylindrical HA microparticles and round nanoparticles around the HA are liposomes; scale bar = 100 nm HA + BTL (left panel), HA + NT (right panel).

**Figure 4:**
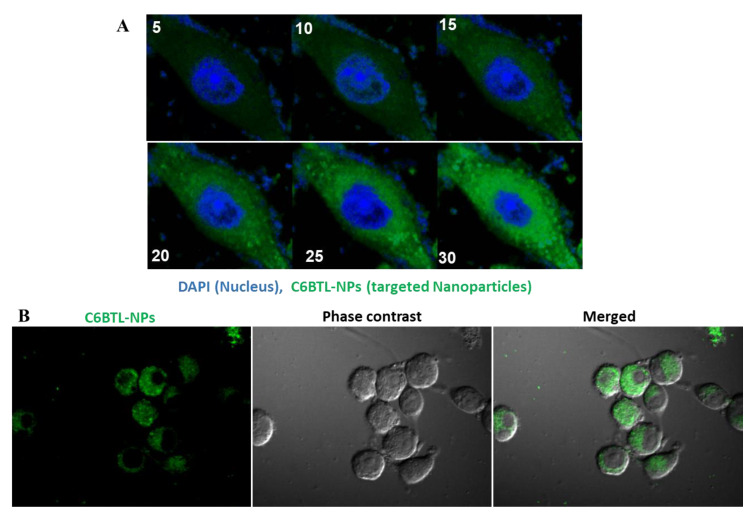
Confocal microscopy of C6BTLs uptake by CD271+BM-MSCs. Cells were incubated with C6BTL followed by live cell imaging to evaluate the time-bound uptake of BTLs by cells. (**A**) RAW 246.7 cells capture every 5 min up till 30 min after the addition of C6BTL. (**B**) CD271+BM-MSCs incubated with C6BTLs, fixed and imaged after 30 min.

**Figure 5:**
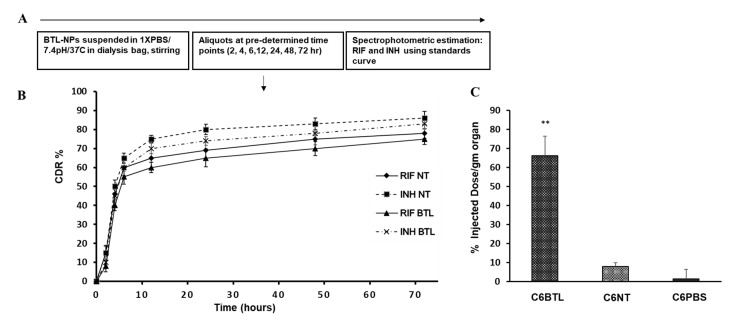
(**A**) Schematic of in vitro drug release. (**B**) Cumulative drug release pattern (CDR%) of targeted (BTLs) versus non-targeted (NT) for INH and RIF. A 500 µL volume of INH- and RIF-encapsulated BTLs and NTs were suspended in dialysis bags and stirred for 72 h in 25 mL of 1× PBS (pH 7.4). At each time point, 500 µL was aliquoted from a 25 mL reservoir for drug release assay. Data represents mean CDR% ± SEM (*n* = 3). (**C**) Tissue uptake of targeted (C6 BTL) versus non-targeted (C6 NT). Data are presented as % of C6 injected recovered per g of organ (organ = pair of tibia and femur). SEM (*n* = 3), ** *p* < 0.001 vs. C6NT.

Alendronate tagging of PEG-PE was carried out for formulating Alen-PEG-PE as illustrated (Supplementary Figure S1). Resulting Alen-PEG-PE was used for the synthesis of BTL liposomes using additional PE-PEG and PC (Figure 1A,B). INH, RIF and Coumarin6 (fluorescent marker C6) were separately encapsulated in BTLs to formulate three separate BTLs: INH BTL, RIF BTL and C6 BTL followed by characterization (Figure 2). C6 BTLs were used for bone affinity experiments (Figure 3), cellular uptake (Figure 4) and tissue uptake studies (Figure 5C). Transmission electron microscopy results showed that all liposomal formulations consist of unilamellar vesicles in the size range of 80 to 110 nm having a spherical morphology. Dynamic light scattering data showed that the mean diameter of the prepared formulations, C6 BTLs, INH BTLS and RIF BTLs, were 109 ± 12.6 nm, 100 ± 16.3 nm and 84 ± 18.4 nm in diameter, respectively. Zeta potential values were negative which facilitates the long circulation of liposomes (Figure 2A–C). Average drug encapsulation efficiency determined for C6 BTLs, INH-BTLs and RIF BTLs were 70% ± 3.7%, 69.5% ± 4.2% and 70.6% ± 4.7%, respectively (Table 1). Non-targeted liposomes exhibited similar size range and drug encapsulation efficiency.

### 2.2. Binding Assay of Alendronate BTL-NPs

Alendronate has a natural affinity toward the calcium component of Hydroxyapatite (HA) crystal. To investigate the affinity of engineered BTLs for BM, bone-chip binding and HA binding experiments were performed. In order to visualize qualitative binding, C6 (a fluorescent marker)-loaded BTLs were used. Alen-tagged BTLs showed a high fluorescence signal (green), whereas the non-targeted liposomes did not show any binding (Figure 3A), proving the high affinity of BTLs for mice bone fragments (femur). In the second binding assay, HA microparticles were allowed to bind with alen BTLs and non-targeted liposomes. TEM imaging results showed that HA binds to alen BTLs with greater affinity while non-targeted liposomes did not show any affinity to HA microparticles (Figure 3B). Overall, these results confirmed that BTLs exhibited a strong affinity to bone HA and therefore may possess bone-homing capabilities.

### 2.3. Cellular Uptake of Alendronate BTL-NPs by CD271+BM-MSCs

To assess the cellular uptake efficiency of BTLs, two different cells were investigated: macrophages (RAW 264.7 cell line murine macrophages) and freshly isolated murine CD271+BM-MSCs. Using live cell imaging, RAW 264.7 cell uptake was observed from 5 min to 30 min after the addition of BTL. Figure 4A indicates the successful uptake of BTLs by macrophages. Next, mice CD271+BM-MSCs were incubated with alen BTLs which exhibited similar uptake patterns showing localization inside the cells at 30 min (Figure 4B).

### 2.4. In Vitro Drug Release Profile of INH and RIF BTL-NPs and Tissue Uptake 

In vitro drug release profile was enumerated by an in vitro dialysis method. The INH-encapsulated alen BTLs and RIF-encapsulated alen BTLs showed sustained slower release till 72 h in comparison to their counterpart non-targeted NPs as indicated by % cumulative drug release pattern (CDR%). Notedly, INH-BTLs showed faster release compared to RIF BTLs (Figure 5A,B). Nevertheless, both targeted NP’s cumulative drug release was slow and sustained. Further, we investigated the bone uptake of BTLs, where mice were injected with C6 loaded alen BTLs, C6 non-targeted (NT) liposomes or C6 PBS. Pairs of femur and tibia were excised followed by C6 extraction with chloroform: methanol. Fluorimetric analysis was carried out for determining the percentage of C6 dose injected, recovered from both bone pairs. We found that there was an approximate eight times increase in the homing of C6BTLs in comparison to C6NT liposomes (Figure 5C) demonstrating the bone-seeking nature of BTL-NPs. 

### 2.5. Efficacy of INH and RIF BTL-NPs in H37Rv Infected Mice

**Figure 6:**
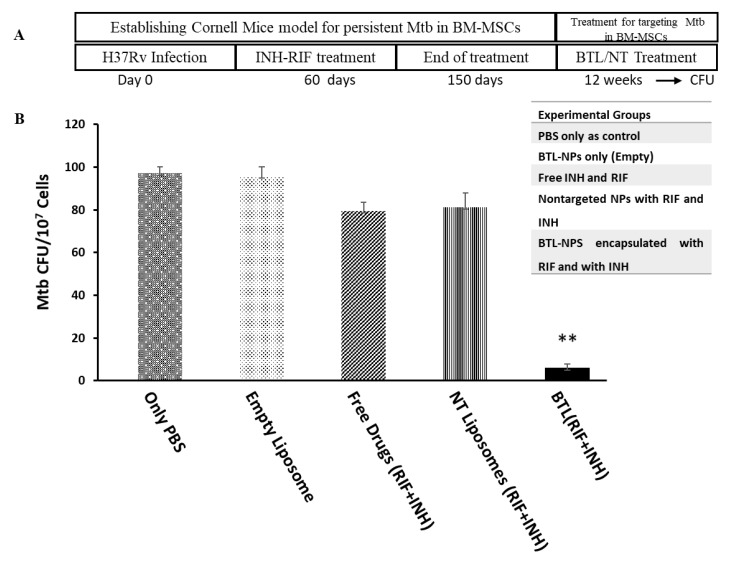
*Mycobacterium tuberculosis* (Mtb) load from CD271+BM-MSCs post-INH and RIF BTLs treatment. (**A**) Schematic of in vivo treatment. (**B**) C57bl/6 mice were infected with H37Rv, administered with standard anti-Tb drugs and next administered with drug-loaded BTL or NTs for 12 weeks/twice a week/intravenously. The effective dosage of INH and RIF was maintained at 4 mg/kg and 3 mg/kg, respectively, for all drug groups. Mtb CFU were enumerated by plating CD271+ BM-MSCs population obtained from immuno-magnetic sorting. *n* = 3, data expressed as mean CFU = +/− SEM per 10^7^ cells. ** *p* < 0.001.

For evaluating the efficacy of INH and RIF alendronate BTLs, standard Cornell model of persistent Mtb was followed (Figure 6A): Mice infected with pathogenic strains of Mtb H37RV were administered standard INH and RIF dosage for 90 days as a result of which all organs except for bone marrow were free of bacilli load [11,13]. After one week of the above regimen, animals were treated with the drug-loaded BTLs/NT NPs for 12 weeks, twice per week, with an effective concentration of INH = 4 mg/kg and RIF = 3 mg/kg. Appropriate controls were used. Animal health and weight were monitored for four months followed by CFU enumeration of CD271+BM-MSCs. A significant reduction of Mtb CFU in CD271+BM-MSCs was observed (Figure 6B). Notedly, Mtb culture from lungs was found negative in all experimental groups. 

**Figure 7:**
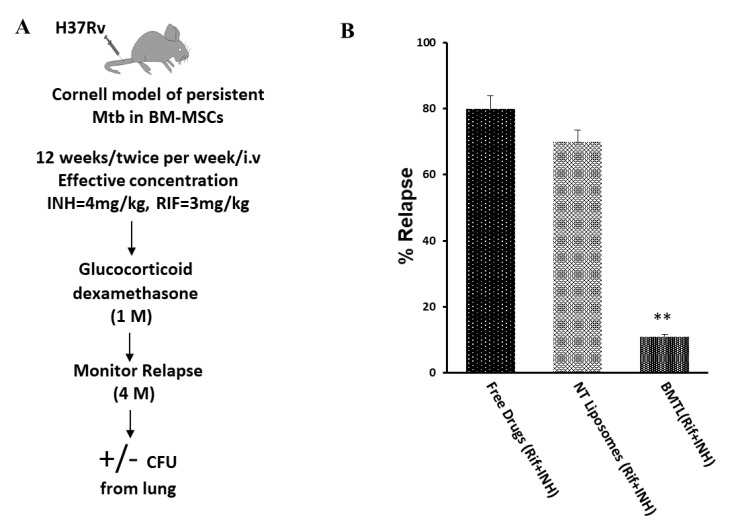
Relapse rate for INH and RIF BTLs versus NT liposomes. (**A**) Schematic of in vivo treatment for relapse. (**B**) Relapse % for targeted liposomes. Mice from each group were monitored for 4 months for signs of relapse. Animals were sacrificed for relapse signs: positive culture from lungs and gross lung pathology/granuloma formation. Data expressed as mean = +/− SEM. ** *p* < 0.001.

Relapse studies were carried out on Mtb-infected mice as illustrated in Figure 7A. Mice were monitored up to four months for relapse post-dexamethasone administration (10 mg/kg/ four weeks). Gross pathology inspection of the lung lobes was carried out for granuloma formation and the remaining portion of the lung was homogenized and cultured to score positive or negative Mtb load (Figure 7). Appropriate controls were included for relapse study viz. only vehicle and empty BTLs. All untreated mice succumbed to death within two months of infection. The relapse rate was 11% for the BTL group while the other experimental groups showed a higher relapse rate (Figure 7).

## 3. Discussion

We and others have shown that CD271+BM-Mesenchymal stem cells are natural reservoirs for dormant Mtb [11,13,14]. In this study, we engineered bone-homing liposomes decorated with Ald and PEG to actively target Mtb in its novel niche. Our BTL-NPs showed significant bone binding capabilities and resulted in the decreased Mtb load of CD271+BM-MSCs. Although bone-homing liposomes have been reported earlier [42,43], this is the first study to formulate and test alen-conjugated PEG liposomes to target BM-resident Mtb. Multiple functions were kept in mind and were incorporated into the engineering of BTL-NP: (1) bone-homing capability, (2) stealth properties by PEG and (3) size. For bone-homing, alendronate, a biphosphate was attached to the liposome surface resulting in high affinity and homing of BTLs to the bone microenvironment. Alendronate belongs to the bisphosphonate family of drugs and is generally used for cancer therapy. Previous studies show that alendronate has no cytotoxic effects and it does not alter the differentiation or self-renewal properties of stem cells residing in the bone marrow [35,43,44,45]. It is known that alendronate treatment can be helpful in the treatment of bone disease, especially bone cancer [46]. Alendronate, 5 or 10 mg/d, may increase the bone mineral density in women (postmenopausal) and men with primary osteoporosis [47]. Alendronic acid, administered at a 70 mg once weekly or 35 mg twice weekly dose, has shown to increase BMD (bone mineral density) as 10 mg/d and is already used clinically in the treatment or prevention of osteoporosis [48]. While alendronate on systemic administration homes to the bone microenvironment, PEG improves the systemic circulation of liposomes [34,49]. High circulation time owing to PEGylation, high bone-homing capability of BTL-NPs due to alendronate and optimal size in our study may have rendered them effective for clearance of CD271+BM-MSCs resident Mtb.

Our in vivo data indicates that BTL-NPs may have increased the available intracellular RIF and INH leading to Mtb clearance inside BM-MSCs. We also speculated that the PEG-modification of BTLs passively increased the bone marrow selectivity by inhibiting the hepatic uptake, resulting in BTL-NPs to target BM macrophages [35,50]. Moreover, it is possible that after getting trapped in macrophages, BTL-NPs gradually get digested by lysosomal enzymes leading to controlled drug release from macrophages to the surrounding BM tissue, further increasing intracellular drug. While MSCs are known to have drug efflux pumps that gradually efflux out drugs/antibiotics to protect the cells from cytotoxicity, our data suggest that the BTL-NPs may be delivering the drugs at a faster pace in comparison to their efflux rate. In aggregate, it is possible that BTL-NPs may increase the available intracellular drugs, rendering them more effective in Mtb killing compared [51] to free drugs or NT NPs. However, we acknowledge that this speculation requires further investigation.

Lung-homing liposomes targeting Mtb lowers treatment duration and hepatotoxicity in pulmonary tuberculosis [40,52,53]. Swami et al. recently showed that PLGA nanocarriers could be used for the targeted delivery of drugs to bone cancer [35]. These two studies taken together led to the hypothesis that BM-homing liposomes could aid in clearing tubercle bacilli present in the BM cells with reduced off-target toxicity. We acknowledge that, although the entire Mtb population from BM cells was not cleared leading to relapse in very few mice, a decrease in relapse rate is of considerable clinical significance. Few Mtb cells remaining in CD271+BM-MSC (Figure 6B) despite BTL treatment, indicate that there might be some subpopulation of Mtb which (a) may be pushed into deeper stage dormancy, (b) may have developed into drug resistance variants or (c) were present in an unreachable compartment of CD271+BM-MSCs. Any of these three possibilities require further investigation as deeper understanding may lead to complete clearance in BM cells. Nevertheless, currently, there is no treatment/drug which targets BM-MSCs resident Mtb and since alendronate is already in clinical use for bone strengthening, alendronate-based bone targeting may be of clinical significance. Once clinically investigated, BTLs may be used to reduce the relapse rate in post-chemotherapy patients. Overall, our study demonstrates that BTL-NPs can be used for the effective delivery of existing anti-TB drugs to bone microenvironment through specially formulated liposome carriers.

## 4. Materials and Methods

### 4.1. Alendronate Tagging of DSPE-PEG

First, 23 mg of DSPE-PEG (2000)-carboxylic acid (1,2-distearoyl-*sn*-glycero-3-phosphoethanolamine-N-[carboxy(polyethylene glycol)-2000]ammonium salt(Avanti lipids polar, Inc., USA, Cat Number 880125P) was dissolved in 5 mL of acetone. Next, 4.2 mg N,N-Dicyclohexylcarbodiimide (DCC) and 2.4 mg N-hydroxy succinimide (NHS) were added for activation overnight at RT. Syringe assisted removal of insoluble by-product (Dicyclohexylurea ) was carried out. Following this drying of lipids was carried out for 2 h through nitrogen. For alendronate tagging, activated lipid and 2 mg alendronate sodium trihydrate (Ald, A-4978, Sigma-Aldrich, St. Louis, MO, USA) were dissolved in a mixture of DMSO and water for 24 h. This was followed by 24 h dialysis against water and subsequent drying under nitrogen. 

### 4.2. Preparation of PEG Liposome and BTL PEGylated Liposome NPs with Ald Tagged PEG-PE and PC (with INH and RIF Encapsulation)

Liposome NPs were prepared as described previously [21,54,55]. In brief, Egg-PC and Egg-PE in a molar ratio of 8:2 were mixed in a 100 mL round bottom flask in chloroform and rotated under vacuum at 37 °C until a thin lipid layer is formed followed by desiccation for 2 h. For alendronate PEGylated liposomes a.k.a. BTLs, PC (2% of total lipids) + Ald-PE-PEG2000 (2.5% of total lipid content) + PE-mPEG2000 (2.5% of total lipid content) ratio was used. The resultant dry lipid was vortexed with PBS until complete lipid dispersion. For RIF-encapsulated BTL-NPs, Rifampicin (M.P Biomedicals, CAS #13294-4-1, Irvin, CA, USA) was dissolved in chloroform, and for INH-encapsulated BTL-NPs, isoniazid (Sigma-Aldrich, CAS #54-85-3, St. Louis, MO USA) was dissolved in saline at appropriate concentrations. Next, liposomal dispersion was centrifuged at 50,000 rpm (60 min) at 4 °C. This step was done to get rid of un-encapsulated drugs. The pellet obtained was resuspended and centrifuged twice to remove the traces of un-encapsulated drugs. For equal size and decreased variation, the final suspension obtained after three washing was extruded sequentially through 3 membrane filters. The liposomal suspension was assayed spectrophotometrically for the presence of RIF and INH, according to the modified method described [56]. Millipore membrane filters used were of Type RA from Millipore Corp., Bedford, MA, USA.

### 4.3. TEM and Size Distribution

The liposomal suspension was diluted in distilled water and placed upon 300-mesh carbon-coated copper grids and air dried for analysis [21,54]. The liposomes were visualized under a transmission electron microscope (JEOL 2100F). Zetasizer Nano ZS (ZEN 3600; Malvern Instruments, Worcestershire, UK) was used to measure NP size and zeta potential. Detailed NP characterization was performed as previously described by Rajendran et al. [55]. Briefly, NPs were diluted 1000-fold in water in order to make homogenous suspension (scatter angle = 90 °C, temperature = 25 °C) and analyzed by placing in a zeta cell (DTS-1060C). 

### 4.4. Drug Concentration Estimation and Encapsulation Efficiency

Drug encapsulated liposomes were lysed by vortexing in 70% ethanol and incubated at 60 °C. Drugs (INH and RIF) were quantified by spectrophotometric absorbance method against a free drug standard curve at 263 nm and 479 nm, respectively [56].

Encapsulation efficiency = (Drug concentration in eluent/drug concentration loaded) × 100

### 4.5. Cellular Uptake by Mouse CD271+ BM-MSCs

Briefly, 2 × 10^3^ CD271+BM-MSCs and RAW 246.7 cells were seeded in 8-well glass-bottom chambered slide (Ibidi) in D-MEM/F-12 medium with GlutaMAX™-I and DMEM (Thermo Fisher Scientific, Cat. Number 10565018, Cat. Number 12100046, Waltham, MA, USA) serum supplemented media, respectively. After 4–5 h of seeding, prior to imaging, Coumarin6 NP (effective C6 concentration = 2 µg/mL) was added. Live cell imaging was carried out to monitor time lapsed uptake of engineered BTL with Coumarin6 using Nikon Camera Nikon Real-Time Laser Scanning Confocal Microscope, Model A1R. Notedly, C6 is a common fluorescent model drug used for tracking NP uptake [57].

### 4.6. NP Binding Assay with Bone Chips

Mice were sacrificed to the excise femur bone. Next, bone chips from femur were washed with 1× PBS, incubated with alendronate BTLs encapsulating Coumarin6 (alen BTL C6) or non-targeted liposomes C6 (NT liposomes C6) for 30 min followed by washing. The whole procedure was carried out in 8-well glass-bottom confocal slides. Lastly, each well was added with PBS for visualization through a confocal microscope (Nikon Camera Nikon Real-Time Laser Scanning Confocal Microscope, Model A1R) for the binding of C6 NPs with mouse femur bone chips obtained [35].

### 4.7. In Vitro Release from BTL-NPs and Tissue Uptake

For in vitro release assay 500 µL of NPs suspension in phosphate-buffered saline (PBS) were added to a dialysis bag (MWCO 12,000–14,000; Sigma-Aldrich, St. Louis, MO, USA). The dialysis bag was placed into 25 mL of PBS (pH 7.4) taken in a dissolution vessel at 37 °C and stirred at 50 rev min^−1^. At periodic intervals, 500 µL samples were aliquoted from the dialysate and then an equal volume of PBS (pH 7.4) was added. INH and RIF were quantified by measuring absorbance against the free drug standard curve at 263 and 479 nm, respectively. The modified procedure described previously [58] was performed for tissue uptake assay. Briefly, after 8 h of administration of C6 BTL, C6 NT or C6 PBS intravenously, femur and tibia pair were excised followed by chloroform: methanol extraction. The same effective concentration of C6 (150 mg/kg) was administered to all three mice groups. Fluorescence of resulting suspension was determined using Cary Eclipse Fluorescence Spectrophotometer (Agilent) in a quartz cuvette at the emission/excitation of 460/525 nm. The concentration of C6 in the suspension was determined by using the standard curve of C6. % injected a dose of C6 = Conc. of C6/Total injected C6) × 100 per g of organ. Data were represented as % dose injected per organ (per organ in biodistribution study = a pair of tibia and femur from single mice). 

### 4.8. In Vivo Administration and Assessment of BTL-NPs Efficacy

Pathogen-free, 6 weeks female C57bl/6J mice were obtained from the National Institute of Nutrition (Hyderabad, India). Animals were housed and fed according to the standard norms of Animal biosafety level 3 (ABSL3) at Jawaharlal Nehru University, New Delhi. All studies were carried out post-approval from Institution of Animal Ethics Committee (IAEC) and the Institution of Biosafety Committee (IBSC) at Jawaharlal Nehru University, New Delhi. 

Mice infections were performed as previously described [13]. Briefly, C57bl/6J female age 6 weeks were infected with H37Rv 2 × 10^6^ CFU through tail vein and followed by anti-TB drug therapy (RIF = 0.1 g/L, INH = 0.25 g/L) for 90 days to achieve a status where only Mtb survives in CD271+BM-MSCs while other organs are rendered sterile [11,13]. This model known as the Cornell model has been known for decades where animals treated with anti-Tb drugs wipe out Mtb from lung, liver and spine rendering them sterile, however for no clear reason the disease is relapsed [7,59,60]. It has been shown in mice that this relapse could be due to Mtb present in a novel host cell namely CD271+BM-MSCs [13]. It is postulated that Mtb mesenchymal stem cells may travel from BM to the lung via blood circulation and may cause a relapse of pulmonary TB [11,13]. We have used this Cornell model for targeting Mtb in BM-MSCs. Animals were treated with various treatments including free drugs or BTL-NPs intraperitoneally. The concentration of INH and RIF remained under the safe limits which are 10 mg/kg (standard) for both the drugs [61]. Briefly, INH and RIF average concentrations fell in the range of 2–2.3 mg/mL and 2.66–3.1 mg/mL for their respective NP suspensions (BTL or NT). All liposomes and free drug solutions were diluted to the effective dosage of 3 mg/kg (RIF) or 4 mg/kg (INH) per mice in 150 µL. To be noted, NPs were freshly prepared for each administration and concentrations of drug in each formulation was determined using a standard curve for each drug as mentioned in method Section 4.4. At the end of the regimen, mice were sacrificed and Mtb CFU from CD271+ BM-MSCs sorted cells were enumerated by plating on 7H11 agar plates supplemented with 10% Middlebrook OADC. 

### 4.9. Enumeration of Mtb CFU from CD271+BM-MSCs

The assay was performed as previously described [11,12,13]. BM cells were aseptically obtained from each group followed by magnetic sorting CD271+ BM-MSCs as described previously [13]. Briefly, an average 4 × 10^7^ bone marrow cells were obtained from a femur pair per mice after RBC lysis. BM cells were then used for performing CD271+ BM-MSCs selection by magnetic sorting (Cat. Number S10467, Life Technologies, Carlsbad, CA, USA; Cat. Number 18554, Stem Cell Technologies, Vancouver, BC, Canada). Sorted cells were lysed followed by plating on 7H11 plates supplemented with 10% Middlebrook OADC. 

### 4.10. TB Relapse Assay

A similar experiment as described in the assessment of BTL-NPs Efficacy (Section 4.8) was carried out. NP treatment regimen was followed by steroid administration (glucocorticoid dexamethasone, intraperitoneal injections of 200 μL of dexamethasone at 10 mg/kg of body weight every 2 days for 4 weeks) and were sacrificed after 4 weeks to look for signs of reactivation. Signs for relapse were (a) granuloma formation in lungs by gross pathology and (b) culture-negative or culture-positive for lungs. Relapse percentage was calculated as follows:

Relapse % = (No. of mice with culture-positive relapse/total No. of mice) × 100

### 4.11. Statistical Analysis

Experimental data were analyzed by GraphPad Prism. Student’s *t*-test was performed to compare two groups. Data are expressed as means = +/− SEM; ** *p* < 0.001.

## Figures and Tables

**Table 1:** Size and drug encapsulation efficiency of BTL-NPs.

Nanoparticle (NP)	NP Size (nm)	% Encapsulation
C6 BTL-NP	109 ± 12.6	70 ± 3.7
INH BTL-NP	100 ± 16.3	69.5 ± 4.2
RIF BTL-NP	84 ± 18.4	70.6 ± 4.7

## Supplementary Materials

The following are available online at https://www.mdpi.com/2076-0817/9/5/372/s1, Figure S1: Schematic for synthesis of Alendronate conjugation of DSPE-PEG(2000) from DSPE-PEG(2000) Carboxylic Acid and alendronate sodium trihydrate.
