# Supplementary material for: Preclinical Evidence of Nanomedicine Formulation to Target Mycobacterium tuberculosis at Its Bone Marrow Niche
Source: Pathogens. 2020 May 13;9(5):372. doi: 10.3390/pathogens9050372 (PMC7281663; doi:10.3390/pathogens9050372)
Supplement: Supplementary file 1 [file pathogens-09-00372-s001.pdf]

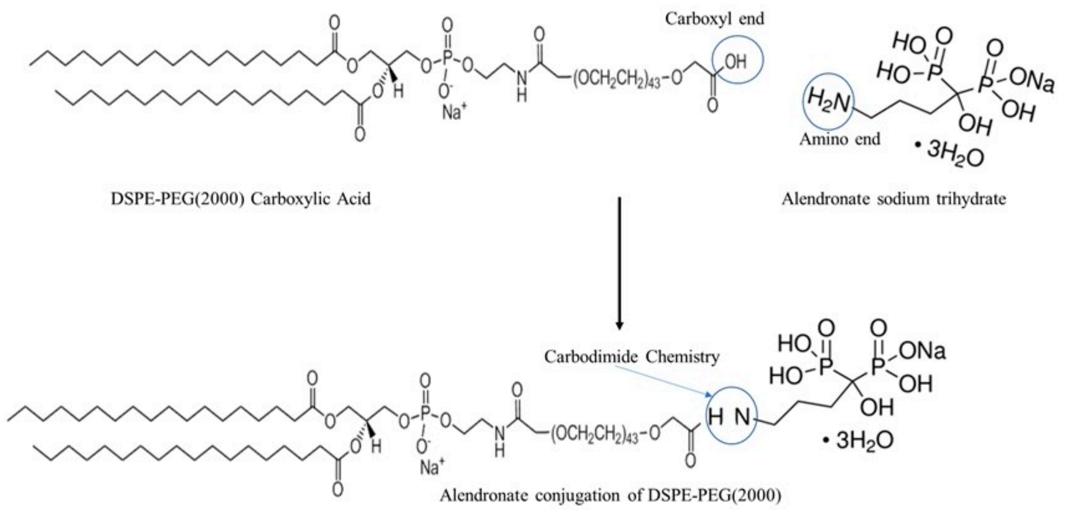

Figure S1. Schematic for synthesis of Alendronate conjugation of DSPE-PEG(2000) from DSPE-PEG(2000) Carboxylic Acid and alendronate sodium trihydrate.
